# Arsenic metabolism in technical biogas plants: possible consequences for resident microbiota and downstream units

**DOI:** 10.1186/s13568-019-0902-6

**Published:** 2019-11-28

**Authors:** Nicolas Weithmann, Stanislava Mlinar, Frank Hilbrig, Samer Bachmaf, Julia Arndt, Britta Planer-Friedrich, Alfons R. Weig, Ruth Freitag

**Affiliations:** 1 Process Biotechnology and Centre for Energy Technology (ZET), University of Bayreuth, Bayreuth, Germany; 2 Department of Environmental Geochemistry, Bayreuth Centre for Ecology and Environmental Research (BayCEER), University of Bayreuth, Bayreuth, Germany; 3 Genomics and Bioinformatics, University of Bayreuth, Bayreuth, Germany

**Keywords:** Anaerobic, Archaea, Biogas, Metal(loid), Methanogenesis, Sewage sludge

## Abstract

The metal(loid) and in particular the Arsenic (As) burden of thirteen agricultural biogas plants and two sewage sludge digesters were investigated together with the corresponding microbial consortia. The latter were characterized by ARISA (automated ribosomal intergenetic spacer analysis) and next generation sequencing. The consortia were found to cluster according to digester type rather than substrate or metal(loid) composition. For selected plants, individual As species in the liquid and gaseous phases were quantified, showing that the microorganisms actively metabolize and thereby remove the As from their environment via the formation of (methylated) volatile species. The As metabolites showed some dependency on the microbial consortia, while there was no statistical correlation with the substrate mix. Finally, slurry from one agricultural biogas plant and one sewage sludge digester was transferred into laboratory scale reactors (“satellite reactors”) and the response to a defined addition of As (30 and 60 µM sodium arsenite) was studied. The results corroborate the hypothesis of a rapid conversion of dissolved As species into volatile ones. Methanogenesis was reduced during that time, while there was no discernable toxic effect on the microbial population. However, the utilization of the produced biogas as replacement for natural gas, e.g. as fuel, may be problematic, as catalysts and machinery are known to suffer from prolonged exposure even to low As concentrations.

## Introduction

Biogas is considered a possible substitute for fossil fuels such as natural gas in a sustainable energy mix. The main components of biogas are CH_4_ and CO_2_. In addition, a few trace compounds are discussed in the context of an industrial utilization, most prominently H_2_S, which is known to harm downstream machinery, and ammonia, which in addition may also harm and inhibit the biogas producing microbial consortia (Rönsch et al. 2016; Yenigün and Demirel 2013). However, the number of elements entering a biogas plant via the substrate and/or various additives is much larger and includes also a wide selection of metal(loid)s. Volatile compounds exist for many of these, yet it is generally assumed, e.g. also by the European Biogas Association, that they do not occur in significant amounts in the produced gas (Svensson 2014). Surprisingly, this includes also As species, even though natural biogas formed in swamps and marshes, but also in rice paddies, has been shown to contain, *inter alia* volatile As species (Jia et al. 2012; Zhang et al. 2016).

Inorganic As compounds are ubiquitous in the environment (Irgolic et al. 1991). The average content of As in the earth crust is 3.4 mg kg^−1^ (Wedepohl 2008). Soil contents may vary widely, e.g., from average values in uncontaminated soils of 5 to 6 mg kg^−1^ (Lepp 1981) to up to 156 mg kg^−1^ in certain farm lands in Bavaria, Germany (Geuß et al. 2011). Artificial fertilizer used in Europe contains on the average 7.6 mg kg^−1^ of arsenate (Nziguheba and Smolders 2008). In Germany, the upper allowable limit in fertilizer is 40 mg kg^−1^, while starting from 20 mg kg^−1^ onward, labeling becomes obligatory (BMJV 2012). Various biological plants have been described to take up arsenate through phosphate uptake channels. This includes a number of energy crops typically used in biogas production, such as corn and barley (Asher and Reay 1979; Jacobs and Keeney 1970; Sadiq 1986).

Most microorganisms have developed some ability to decontaminate their habitats by removing toxic substances such as heavy metals and metalloids. A frequent strategy is alkylation, in particular methylation, which ultimately renders the compounds volatile. For As methylation, the Challenger pathway (Challenger 1945) is used by many organisms, including humans, algae, and yeasts. This pathway eventually produces volatile trimethyl arsine (TMA) via a series of reduction and methylation/oxidation steps involving also mono and dimethyl arsine (MMA, DMA), with S-adenosyl methionine (SAM) typically acting as donor of the methyl groups. However, experiments with fungi showed that As methylation in microorganisms can also follow significantly more complex mechanisms than the simple Challenger mechanism, see Bentley and Chasteen (2002) for a review. Moreover, studies with single strain cultures have demonstrated that the formation of methylated As species occurs in direct competition to methanogenesis (Thomas et al. 2011). Mono-, di- and tri-methylated arsenates and arsenites were identified as intermediates in these experiments, with the alkylated pentavalent arsenates found mostly in the liquid phase, whereas volatilization was accompanied by a reduction to the alkylated arsenites and arsines. In addition to methylated As species, arsine (AsH_3_) itself can also be formed by microbial hydration of the As species. Finally, certain chemoautotrophic bacteria may also use the oxidation of arsenites to arsenates to gain energy (Zhang et al. 2015; Oremland et al. 2002).

The ability of microorganisms to form methylated As compounds has been shown under both aerobic and anaerobic conditions. Michalke et al. (2000) demonstrated As methylation for sewage sludge communities in a municipal wastewater treatment plant. Mestrot et al. (2013) challenged laboratory scale biogas digesters with methylated arsenate species in order to document the volatilization kinetics. The most abundant volatile species (over 80%) found in these experiments was TMA. Moreover, some authors have presented evidence that volatile As species in natural gas may cause problems in technical appliances. Xu et al. (2015) mentioned an international safety standard for the As concentration in natural gas of 62.5 ng NL^−1^, as higher levels presumably already pose a threat to machinery. Delgado-Morales et al. (1994) described blocked valves and pipelines in a New Mexican gas field caused by the formation of solid As compounds, later identified as tri- and dialkyl arsine sulfides, while others reported on catalyst failure due to As poisoning (Merryfield et al. 1985).

In this study we investigated metal(loid)s found in agricultural biogas plants and sewage sludge digesters and in particular the production of As species under anoxic conditions in these plants. Effects on the microbial consortia and the biogas production process are evaluated and first conclusions are drawn concerning possible consequences for the utilization of the produced biogas as fuel.

## Materials and methods

### Materials

Standard chemicals, biologicals, and supplements, as well as plastic materials and culture flasks (glass bottles) were from established suppliers and used as received. High quality water was prepared by a Millipore unit.

### Technical plants

Thirteen continuously operated agricultural biogas plants, covering a range of substrate mixes and fermenter types were investigated as well as the sludge digesters of two sewage plants, Table 1. With the exception of plants #4 and #9, the biogas plants were mechanically stirred, continuously operated single (fermenter) or two stage (fermenter-post digester) reactor set ups, fed exclusively with agricultural substrates (mostly energy crops, manure, see Table 1 for details). In case of the two stage reactor set ups, two biogas reactors were operated in sequence. Plant #4 was a non-stirred, discontinuous box fermenter (“garage” reactor type), while mixing and flow in plant #9 were gravity driven. The investigated sewage sludge digesters were both integrated into standard municipal wastewater treatment plants. All biogas plants were approved and certified for electricity production and operated according to standard industrial procedures concerning substrate preparation, reactor feeding and residence times and converted the produced gas into electricity/heat energy. The operating temperatures in the reactors fluctuated between 42 and 48 °C depending *inter alia* on the external temperatures.

**Table 1:** Overview of the investigated technical plants

Plant #	Type	Substrate	Archaeal cluster	Bacterial cluster
*1 (agricultural biogas plant)*^*a*^	*Fermenter—post digester*	*A B C*	*A2*	*B2*
2 (agricultural biogas plant)	Single stage fermenter	A B C E	A1	B1
3 (agricultural biogas plant)	Fermenter—post digester	A D	A2	B2
4 (agricultural biogas plant)	Box digester	A C	A2	None
*5 (agricultural biogas plant)*	*Fermenter*—*post digester*	*A E*	A3	B2
6 (agricultural biogas plant)	Single stage fermenter	A B	A1/A3	B1
*7 (agricultural biogas plant)*	*Fermenter*—*post digester*	*A C E*	A3	B2
8 (agricultural biogas plant)	Fermenter—post digester	A B E	A1	B2
9 (agricultural biogas plant)	Single stage fermenter	B F	A1/A2	B1
10 (agricultural biogas plant)	Fermenter—post digester	A B	A2	B2
11 (agricultural biogas plant)	Fermenter—post digester	A B	A3	None
12 (agricultural biogas plant)	Fermenter—post digester	A C E	A3	B2
13 (agricultural biogas plant)	Fermenter—post digester	B C D	A3	B2
*14 (sewage sludge digester)*^a^	*Single stage digester*	*G*	None	None
15 (sewage sludge digester)	Single stage digester	G	None	None

With: A—energy crops, B—liquid manure, C—solid manure, D—landscaping materials, E—grains, F–fruit peelings, G—sewage sludge

Italic print: plants selected for analysis of As species in the liquid and gaseous phase

^a^Plants selected as basis for the satellites

Samples for metal(loid) quantification in the liquid phase were taken from a suitable outlet pipe of the reactors (after a certain amount of slurry had been discharged) and filled in 1 L PE-bottles. In case of the two-stage plants, only the post digesters were sampled. In case of the box digester, liquid samples were taken from the percolate. Samples were stored at 4 °C in the dark. Samples for the quantification of redox sensitive As species were placed in 300 mL septum-sealed flasks and the headspace was immediately flushed with N_2_. The sample flasks were covered with aluminum foil to avoid exposure to light and then stored in a glovebox (atmosphere: 95% N_2_, 5% H_2_). For biological analysis, 10 mL aliquots of slurry were collected and stored at − 80 °C until extraction of the DNA. To enable a direct comparison, the measured metal(loid) concentrations were normalized to the slurry’s dry weight. For this purpose, 10 g of the homogeneously mixed slurry were dried in triplicate at 105 °C in a drying cabinet until the weight became constant.

For the sampling of volatile As species (only plants #1, #5, #7, #14), 1 L gasbags (Tedlar PLV, Sigma-Aldrich, St. Louis, USA) were connected using viton tubing (ISO-VERSINIC, VWR International, Darmstadt, Germany) to the gas sampling bypasses of the respective main biogas pipes. In case of plant #14, the gasbags were filled passively by the overpressure in the digester, in case of plants #1, #5, and #7 the gas flow was enforced by applying vacuum. To avoid exposure to light, the filled Tedlar^®^ bags were placed in dark plastic bags and stored at 5 °C until analysis. An additional 1 L Tedlar^®^ bag was filled at each biogas plant for the determination of the major gas compounds (percentage of CH_4_, CO_2_).

### Laboratory scale reactors (satellites)

Laboratory scale “satellite reactors” were 1 L glass bottles (“Schott bottles”, DWK Life Sciences, Wertheim, Germany) with screw caps. The satellites were operated with slurry taken directly from two of the technical plants (#1 and #14). The slurry was transported in isolated containers in less than 1 h to the laboratory. The screw caps of the Schott bottles were equipped with three outlet pipes closed by shut-off-valves (neoLab GmbH, Heidelberg, Germany) for sampling and spiking with arsenite (see below). Before starting the experiments, bottles were preheated to approximately 45 °C and flushed with N_2_ for one minute. Then 800 mL of sludge (2.7% dry weight) were added in case of plant #14. In case of plant #1, 600 mL (6.5% dry weight) of sludge were mixed with 200 mL of oxygen-free water in order to facilitate stirring with the magnetic stirrer bar. The reactors were placed in an incubator for temperature control (48 °C for plant #1, 42 °C for plant #14, to mimic conditions found at the corresponding technical plant) and the contents were subsequently stirred for 5 min (100 rpm) once per hour throughout the run of the experiment. Experiments were carried out in duplicates. Different amounts of arsenite (0, 30, and 60 µM) were added 24 h after the start of the satellites by means of a 10 mM NaAsO_2_ solution. Any As being present in the sludges prior to this addition was not quantified. Liquid samples were taken from the satellite reactors using rhizon samplers (MOM 5 mm, Eijkelkamp, Giesbeek, The Netherlands), which were connected by syringe to an evacuated glass vessel (50 mL). For gas sampling, one of the satellite outlets was connected by viton tubing (ISO-VERSINIC, VWR International, Darmstadt, Germany) to 1 L gas bags (Tedlar PLV, Sigma-Aldrich, St. Louis, USA). To document the total biogas production in the satellites, the produced volume of gas between measurements was determined by water volume displacement in over-turned measuring cylinders.

### Analysis of the slurry phase

For the determination of the total amounts of the investigated metal(loid)s in the technical plants, 4 mL of H_2_O_2_ (30% (v/v)) were added to 0.5 g of homogeneously mixed slurry. The sample vessels were closed tightly and stored over night at 4 °C. The next day 3 mL of HNO_3_ (65% (v/v)) were added and the mixture was extracted by microwave digestion (45 min at 220 °C, MARSXpress). All extractions were carried out in triplicate. Extracts were filtered (0.2 μm, cellulose acetate) and stored at 4 °C. The elements Cr, Co, Ni, Se, Rb, Sr, As, Mo, Cd, Sb, Ba, Pb, Bi, U, Mn, Cu, Zn, Al, and Fe were quantified using an X-Series 2 Inductively Coupled Plasma Mass Spectrometer (ICP-MS) (Thermo Scientific, Germany). To account for possible matrix effects, three additional sample aliquots were analyzed after spiking with defined amounts of each analyte.

For the analysis of the dissolved As species, samples were acidified with 6 M HCl and subsequently extracted (1 g of sample digested for 90 min at 95 °C in a heating block). Samples from technical plants were afterwards filtered through cellulose filters (45 µm). Arsenite, arsenate, and methylated As species were quantified by IC-ICP-MS (ion chromatography coupled ICP-MS, Thermo Scientific, Germany). To account for possible matrix effects, additional sample aliquots were spiked with known concentrations of the As species of interest prior to the analysis.

For the analysis of the dissolved volatile fatty acids in the slurries, 500 µL of slurry were acidified with 2 vol % H_3_PO_4_ (85%) to a pH < 2 and centrifuged for 15 min at 13,000 rpm (16,089×*g*). The supernatant was analyzed by gas chromatography (6890 N GC-system, Agilent, Santa Clara, USA) equipped with a FID detector and a FFAP-capillary column (Optima FFAP, Macherey–Nagel, Düren, Germany, 30 m × 320 µm × 0.25 µm) using the following temperature profile: 85 °C for 2 min, heating to 160 °C (10 °C min^−1^), 1 min at 160 °C, heating to 200 °C (35 °C min^−1^), 3 min at 200 °C. Helium was used as carrier gas at a flow rate of 3.0 mL min^−1^. Calibration was by external acetic, propionic, and butyric acid standards (Sigma-Aldrich, St. Louis, USA), recovery was calculated by use of an internal 2-ethyl butyric acid standard (VWR International, Darmstadt, Germany).

All analyses were carried out in triplicate unless otherwise indicated.

### Analysis of the gas phase

Volatile As species were analyzed by automated cryotrapping/cryofocusing gas chromatography (30 m capillary column, 0.32 mm inner diameter, 4 µm film thickness; Rxi-1MS, Restek, USA) coupled to an electron impact mass spectrometer (Varian CP-3800 with Varian Saturn 2000, Varian, USA) with split to an atomic fluorescence analyzer (AFS; P.S. Analytical; equipped with a superlamp, wavelength: 193.7 nm) as described previously (Arndt et al. 2017). For analysis, 50 mL of the gas collected in the Tedlar bags were diluted 1:10 with N_2_. Syringes were pre-flushed with N_2_ to assure the absence of O_2_ prior to sample dilution. Calibration standards for the volatile As species were prepared as previously described (Ilgen and Huang 2013) and kindly provided by the Bayreuth Center of Ecology and Environmental Research, University of Bayreuth. To approximate the matrix of the biogas samples, the standards (2 ng L^−1^ per volatile As species) were prepared freshly before every measurement in a 3: 2 mixture of CH_4_ and CO_2_ diluted 1: 10 with N_2_.

CH_4_-concentrations in the gas samples were determined by gas chromatography (6890 N, Agilent Technologies, Santa Clara, USA) using a packed carbon column (ShinCarbon ST, Restek, Bad Homburg, Germany, 2 m × 1 mm). Helium was used as carrier gas (flow rate of 20 mL min^−1^). The following thermo profile was used: 40 °C for 3 min, heating to 115 °C (8 °C min^−1^), 2 min at 115 °C. The samples for the analyses were taken from the gas bags by an air-tight gas syringe (SGE Analytical Science, Melbourne, Australia).

### Analysis of microbial community

The microbial community structure in all investigated plants was analyzed by automated ribosomal intergenic spacer analysis (ARISA) and 16S rDNA amplicon sequencing. ARISA of bacterial and archaeal communities was as previously published (Weithmann et al. 2016) based on the original protocol by Fisher and Triplett (1999) modified by Weig et al. (2013) using 10 ng of DNA. Ribosomal intergenic fragments were amplified from eubacteria using primers ITSF and ITSReub (Cardinale et al. 2004) and from methanogenic archaea using primers 16S-RIS-M and 23S-RIS-M (Ciesielski et al. 2013). All primers were from biomers.net GmbH (Ulm, Germany). The forward primers were labeled with fluorescent dyes BMN-6 (ITSF) and BMN-5 (16S-RIS-M), to allow parallel detection of eubacterial and archaeal DNA fragments. PCR amplification products were mixed with the MapMarker size standard (50–1200 bp, Bioventures Inc., Murfreesboro, TN, USA) and separated by capillary electrophoresis (GenomeLab GeXP Genetic Analysis System; AB Sciex Germany GmbH, Darmstadt, Germany) using an optimized protocol for long DNA fragments as recommended by the manufacturer. Electropherograms were analyzed using the Genemarker v1.95 software (SoftGenetics, State College, PA, USA). Eubacterial and archaeal fragments were scored and binned from 180 to 893 and from 530 to 893 bp, respectively, and the resulting peak intensity matrix was used for statistical analyses. Procedures and tools for the statistical analysis of ARISA signatures were all embedded in the Primer v7.0.8 and Permanova + addon v1.0.5 software (both from PRIMER-E Ltd., Lutton, United Kingdom). Raw intensity data were first normalized by square root transformation and resemblance matrices were calculated using the Bray–Curtis similarity coefficient. Principal coordinate analyses (PCoA) were conducted separately for methanogenic archaea and for eubacteria. One-way analyses of similarity (ANOSIM) were obtained by using the various substrates of the biogas plants as pairwise factors.

For sequencing, PCR amplification of the 16S rDNA fragments using inline barcodes preceding the 16S-specific primer sequences and high throughput sequencing was conducted using two independent metagenomic DNA replicates produced by LGC Genomics GmbH (Berlin, Germany) as described in Weithmann et al. (2016). Post-processing of the raw data (300 bp paired-end reads, Illumina MiSeq V3) was also performed by LGC Genomics GmbH and included the following steps: demultiplexing of all libraries using Illumina’s bcl2fastq 1.8.4 software, sorting of reads by amplicon inline barcodes to distinguish independent samples, clipping of sequencing adapter remnants from all reads, amplification primer detection and clipping. The resulting 16S rDNA sequences were processed with Qiime2 Version 2017.12. In brief, demultiplexed NGS reads were error corrected using the DADA2 algorithm and taxonomy analysis were performed using taxonomic classifiers, that were trained on the Greengenes 13_8 OTUs (97%) trimmed with the PCR amplification primers. Details of the Qiime2 scripts are embedded in the Qiime2 output files available as Additional file 1; the use of these Qiime2 files is explained at view.qiime2.org.

## Results

### Microbial community structures in the technical plants

**Fig. 1:**
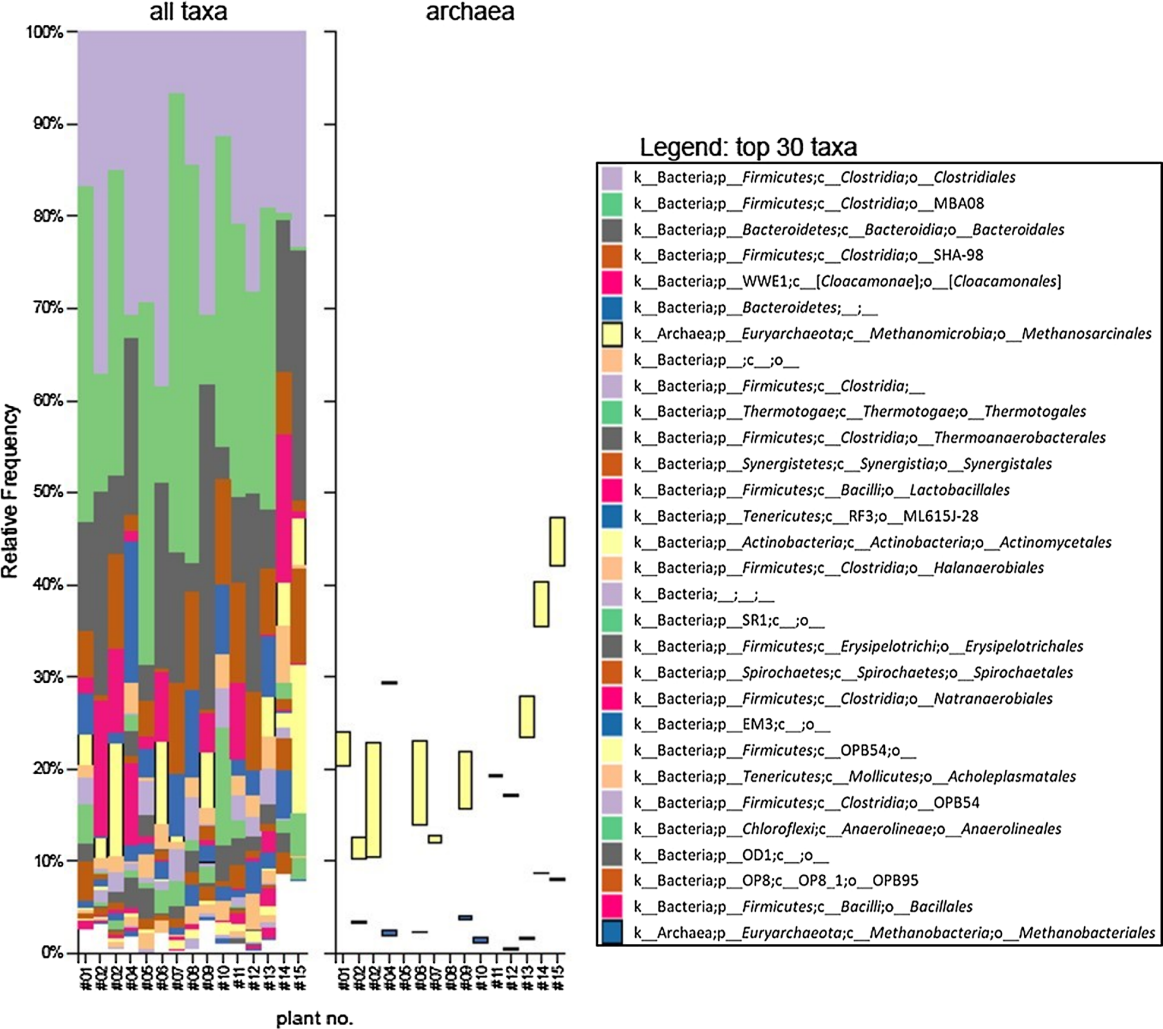
Taxonomic classification of the 16S amplicon reads obtained from metagenomic DNA of biogas plants and sewage sludge digestors. The relative frequency (in decreasing amounts) of taxonomic groups at the level of “order” is shown for all taxa at the left, and for archaea at the right. Only the most abundant 30 taxonomic groups averaged over all samples are shown; the full dataset is available as Additional file 2. x-axis: plant number as assigned in Table 1; y-axis: relative frequency of taxonomic orders

The taxonomic distribution of the organisms in the investigated biogas and sewage sludge digester plants is shown in Fig. 1 for both bacteria and archaea. The relative frequency (in decreasing amounts) of taxonomic groups at the level of “order” is shown for all taxa on the left and for archaea on the right side of that figure. Only the most abundant 30 taxonomic groups (together representing > 95% of the total population in case of the biogas plants and > 90% of the total population in case of the sewage sludge digesters) are shown averaged over all samples; the full dataset is available in Additional file 2: Fig. S1.

Whereas relative abundances and diversities differed somewhat between the plants, representatives from orders belonging to the class of *Clostridiae* dominated in most plants followed by orders from the class of *Bacteroida*. Only in one of the sewage sludge digesters (#15) did *Actinobacteria* dominate. In addition, representatives from all four established classes of methanogenic archaea, namely *Methanobacteria*, *Methanococci*, *Methanomicrobia*, and *Methanopyri* were found in the plants. However, only the *Methanomicrobia* occasionally presented more than 1% of the total population, while *Methanococci* and *Methanopyri* occurred only occasionally. Amongst the *Methanomicrobia*, the communities in most of the investigated plants were dominated by the order of *Methanosarcinales* (often acetoclastic). In addition, hydrogenotrophic *Methanomicrobiales* from that class were found. Finally, most plants also contained significant numbers of *Methanobacteriales* (*Methanobacteria*, typically hydrogenotrophic).

**Fig. 2:**
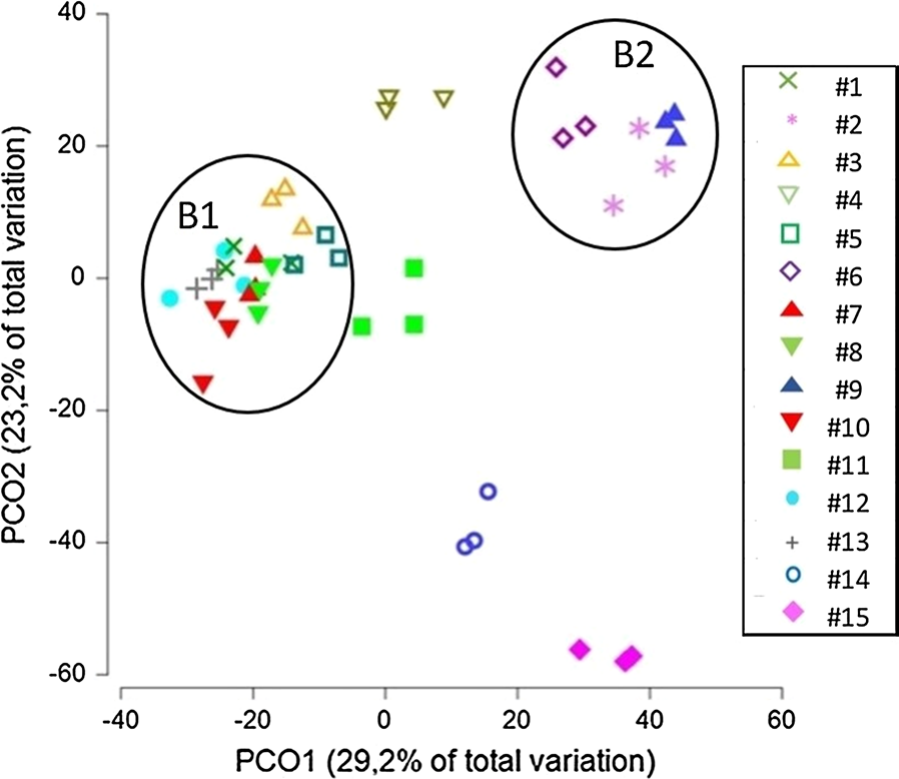
Principal coordinates analysis of the ARISA-fragment patterns obtained for the investigated technical plants (numbering as assigned in Table 1). Three samples per plant were processed and analyzed independently. Bacterial clusters 1B and 2B are indicated by circles

Next, the polymorphism of the SSU and LSU intergenic spacers was used to quickly compare microbial community patterns and dynamics by ARISA (automated ribosomal intergenetic spacer analysis). Using two specific ARISA primer pairs, eubacterial and archaeal communities could be analyzed independently. Not surprisingly in view of the sequence data presented above, the number and diversity of the eubacterial ARISA fragments was much higher than that of the archaeal ones and their patterns showed more pronounced differences between plants. A principal coordinate analysis (PCoA) of the bacterial communities was carried out based on the corresponding ARISA fragments, Fig. 2. For each plant three independent DNA preparation/analyses were done in parallel, the results of which appear in close proximity in the PCoA-plot, demonstrating an acceptable reproducibility of the results. Both sewage sludge digesters (plants #14 and #15) show distinct communities, which differ from each other but also from those of the biogas plants. Among the biogas plants, some clustering can be observed. Three of the plants (#6, #2, #9) form a cluster (B1) in the upper right-hand corner of the PCoA-plot. A second cluster (B2) is seen on the left-hand side in the middle of the plot, which is formed by eight of the biogas plants (#1, #3, #5, #7, #8, #10, #12, #13). The eubacterial communities of biogas plants #4 and #11 show some distance to that of all others, although plant #11 is positioned in proximity to cluster B2.

The plants forming cluster B1 were all single stage fermenters without post digester, whereas the isolated plant # 4 was the only box fermenter (high solids content, no mixing during fermentation) included in our investigation. The communities forming cluster B2 were all from the post digester of the two-reactor plants. Plant #11, which is positioned in close proximity to cluster B2, is also a two-reactor plant. For reasons of accessibility, only the post digesters were sampled for the two stage plants.

In case of the archaea, on the average 9 distinct ARISA fragments were found per plant. The highest diversity/number of fragments (12) was found in plant #10. One of the sewage sludge digesters (#15) contained only a single archaeal fragment type, whereas the second investigated sewage sludge digester (#14) contained 10. In the PCoA of the archaeal fragments, three clusters, A1–A3, were found, as well as two isolated communities, Table 1. Most plants (6) were found in cluster A2, these tended to be of the fermenter/post-digester type, i.e. to also cluster in B2. Four plants formed cluster A1, these tended to appear also in cluster B1, i.e. the cluster formed by the one stage fermenters. The remaining biogas plants formed cluster A3. At least two of the plants (#6 and #9) could not be clearly assigned to one of the archaeal clusters. The archaeal community structures of the sewage sludge digesters were again distinct from each other and different from those of any of the biogas plants.

A correlation of the community structures with the substrate mix or process parameter other than the reactor design was not discernable in the PCoA. However, a pair-wise ANOSIM analysis of the substrate mixes in respect to bacterial and archaeal community structures (significance level: 5%) showed that some substrate pairs differentiate between both archaea and bacteria, while others were significant for bacteria or archaea.

### Metal(loid) concentrations in the plants

Metal(oid)s in the biogas plants and sewage sludge digesters were analyzed comprising the following 19 elements: Cr, Co, Ni, Se, Rb, Sr, As, Mo, Cd, Sb, Ba, Pb, Bi, U, Mn, Cu, Zn, Al, and Fe, Table 2. In this context, As, Sb, Bi, and Se were of particular interest, since these elements are known candidates for volatilization via methylation, while at least As, Sb, and Bi are cytotoxic. With the exception of Se, the highest metal(loid) concentrations were found in the sewage sludge digesters. Se is required for the production of amino acids such as selenium cysteine or selenium methionine, which some methanogens need to oxidize H_2_. To avoid limitation, feeding Se at concentrations between 0.2 and 2.0 mg kg^−1^ of dry weight is recommended for biogas plants and in most of the investigated plants concentrations in the recommended range were found.

**Table 2:** Metal(loid) concentrations found in the investigated plants, normalized to the slurries dry weight [units: mg kg^−1^dry weight]

Plant	#1	#2	#3	#4	#5	#6	#7	#8	#9	#10	#11	#12	#13	#14	#15
Cr	5.6	6.8	12.6	7.9	3.8	6.7	3.2	2.6	5.4	2.9	3.9	3.7	8.6	56.7	85.4
Co	1.8	1.4	1.8	5.0	5.3	1.0	1.7	2.8	2.1	0.8	1.4	1.2	2.5	6.7	8.9
Ni	5.0	6.8	12.0	26.4	9.9	2.8	11.6	4.6	8.3	3.8	4.6	4.6	16.3	24.2	47.7
Se	1.4	n.d.	8.0	n.d.	7.6	0.9	0.3	2.9	8.6	2.4	5.3	n.d.	1.2	0.8	3.0
Rb	46.9	55.6	44.3	119.0	17.4	18.5	31.4	22.4	52.6	16.1	22.3	27.3	57.1	15.3	17.7
Sr	79.2	53.9	85.6	39.7	29.0	32.3	37.0	25.9	80.5	13.2	29.8	42.0	68.3	128.5	73.3
As	1.2	1.5	2.7	2.6	2.3	1.1	2.4	1.4	2.6	1.4	2.7	2.0	3.7	13.8	20.6
Mo	7.3	3.7	3.3	6.7	2.1	1.2	3.5	2.9	1.9	1.7	1.9	3.3	4.6	6.3	6.3
Cd	0.4	0.3	0.5	0.4	0.2	0.2	0.4	0.4	0.2	0.4	0.2	0.2	0.5	1.4	1.2
Sb	n.d.	0.1	n.d.	n.d.	n.d.	n.d.	0.1	0.1	0.2	0.1	0.2	0.2	n.d.	4.3	6.4
Ba	107.5	64.8	71.3	36.2	23.6	39.8	62.2	22.0	47.1	13.4	35.8	49.0	73.2	298.6	165.1
Pb	1.9	2.2	3.3	2.2	0.9	1.0	2.3	3.7	1.6	1.6	1.4	1.7	26.5	35.7	27.7
Bi	n.d.	n.d.	n.d.	n.d.	n.d.	n.d.	n.d.	0.1	1.7	n.d.	0.2	n.d.	0.1	2.5	4.2
U	0.1	0.2	0.2	0.1	0.1	0.2	0.1	0.1	0.3	0.1	0.1	0.4	0.1	5.3	2.9
Mn	247.2	202.2	392.3	8.6	248.1	361.7	279.5	225.3	501.6	179.4	220.0	371.0	263.8	322.1	370.7
Cu	81.3	28.1	55.7	6.2	30.5	41.7	26.9	73.9	57.9	23.7	24.4	54.6	285.4	192.3	192.4
Zn	259.9	140.2	627.0	15.7	196.6	363.5	213.2	219.7	283.1	134.9	138.9	263.5	524.5	893.5	647.4
Al	1462.5	2414.1	2592.7	618.5	528.4	777.1	647.1	1045.3	3130.0	782.1	1234.4	1845.1	2579.1	25,407.4	13,764.8
Fe	1952.4	2942.2	3217.1	2373.5	5190.3	1364.4	3384.2	2135.2	2484.7	2221.2	3933.1	3143.8	5228.9	23,877.5	31,760.2

*n.d.* not detected

Digestates, in particular those from agricultural biogas plants, are often used as organic fertilizers. In this case the allowable upper concentration of some of the metal(loid)s per kilogram of dry weight is limited. For example, in Germany the following upper limits exist according to current regulation: Pb 70 mg kg^−1^, Mo 10 mg kg^−1^, As 10 mg kg^−1^, Co 50 mg kg^−1^, Ni 42 mg kg^−1^, Cu 40 mg kg^−1^, Zn 150 mg kg^−1^, and Cr 60 mg kg^−1^ (BMJV 2012). Most of the plants included in this investigation were of no concern in this regard. One exception was the sewage sludge digester #15, where the values for Cr and Ni as well as for As were surpassed. In the other sewage sludge digester (#14) only the As value would have been problematic in a fertilizer. However, both wastewater plants test the digestates from their sewage sludge digesters prior to further processing (composting). If the threshold values are surpassed, residues are dried and burnt. In several of the biogas plants the threshold level of Cu was surpassed, while in nearly all plants the level of Zn was higher than permissible for fertilizer. In some of the investigated agricultural biogas plants comparatively high As concentrations were found. The highest value, 3.7 mg kg^−1^ of dry weight, was found in biogas plant #13, i.e. a plant using manure together with land scaping materials as substrate, rather than energy crops. Most other biogas plants contained between 1 and 3 mg of As kg^−1^ of dry weight. Sb was not detected in most cases, while significant amounts of Bi were found in plant #9 (1.8 mg kg^−1^ of dry weight). Similar to the situation in case of the substrate mix, it was not possible to establish a correlation between the microbial community structures and the metal(loid) contents or distributions in the various plants.

### Arsenic speciation in the liquid and gaseous phase

**Fig. 3:**
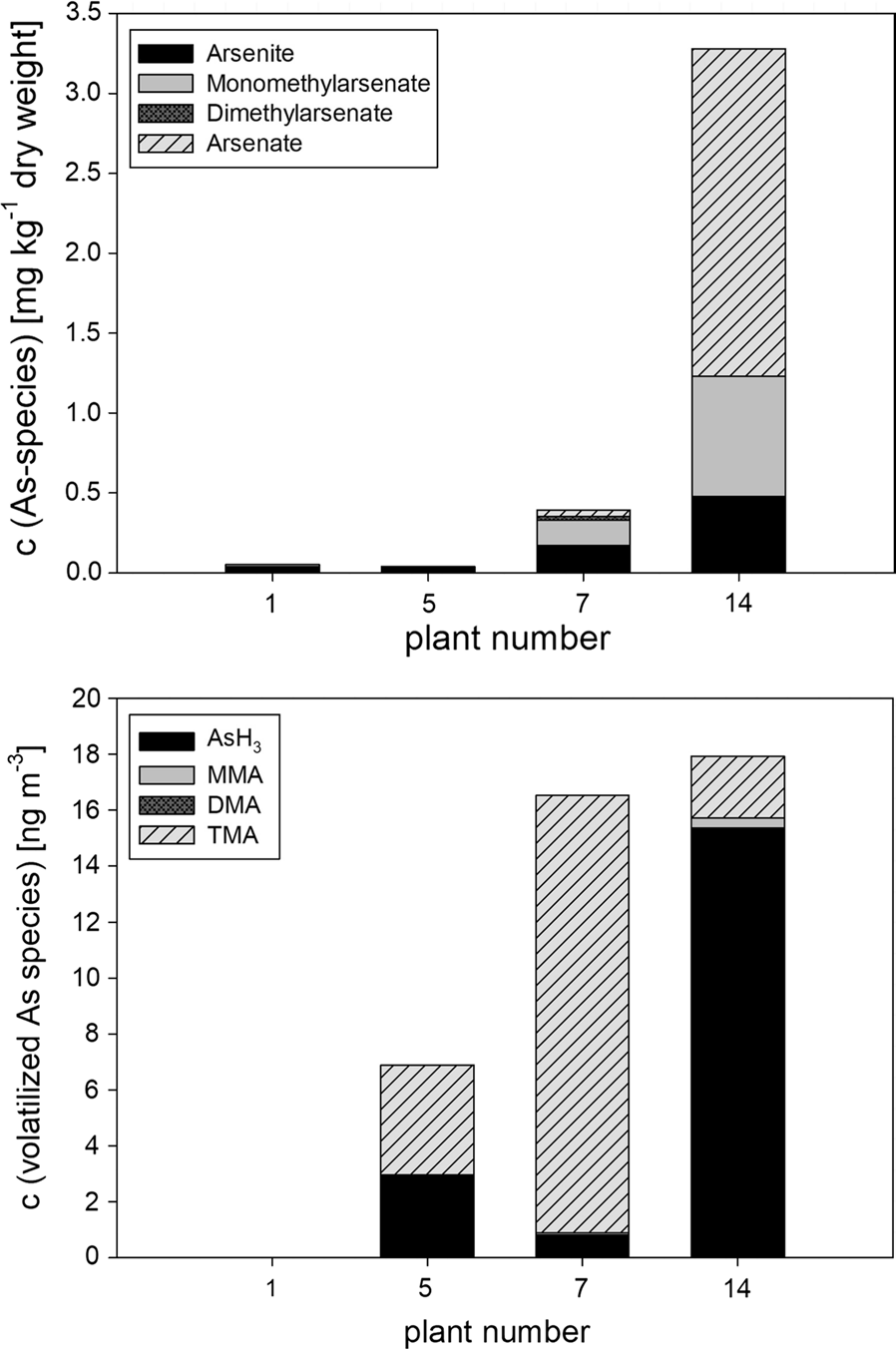
Quantification of the As species in the liquid phase (top) and the produced gas (bottom) of four selected technical plants. The x-axis assigns the plants (numbering as indicated in Table 1). In the liquid phase, only the dissolved (ionic) species were analyzed. Volatile species were only analyzed in the gas phase

In order to gain insight into the linkages between methanogenesis and As metabolism, the major As species in the liquid and the gas phase were analyzed for three of the biogas plants (#1, #5, #7) and one of the sewage sludge digesters (#14). All three biogas plants belonged to the main bacterial cluster B2. With 1.2 mg As kg^−1^ of dry weight, plant #1 had shown the lowest total As concentration of all investigated plants. Plants #5 and #7 were in the middle range (2.3 and 2.4 mg kg^−1^ of dry weight, respectively), while a comparatively high total concentration of As (13.8 mg kg^−1^ of dry weight) had been determined for the sewage sludge digester, plant #14. Results are summarized in Fig. 3.

In all reactors, arsenite was found in the liquid phase. Reactors #7 and #14 contained in addition methylated arsenates as to be expected for a conversion according to the Challenger pathway (Challenger 1945), whereas the sewage sludge digester (plant #14) contained surprisingly also large amounts of unmethylated arsenate. In spite of the fact that it contained similar amounts of total As per kg of dry weight as plant #7 (see Table 2), only traces of arsenite were found in the liquid phase of plant #5; in addition no other dissolved As species could be detected. By comparison, up to 0.17 mg kg^−1^ dry weight of arsenite and 0.16 mg kg^−1^ dry weight of monomethylarsenate were found in the liquid phase of plant #7.

Except for the gas from plant #1, in which no volatile As species could be detected, the gas from the other plants contained mainly AsH_3_ and TMA. This is in accordance with an active methylation along the Challenger pathway. However, whereas the gas from plant #5 contained roughly equal amounts of the two As compounds (3 ng m^−3^ AsH_3_ and 4 ng m^−3^ TMA), the gas of plant #7, where the liquid phase was dominated by arsenite and monomethylarsenate, contained mainly TMA (16 ng m^−3^). The gas from the sewage sludge digester (#14) contained mainly AsH_3_ (16 ng m^−3^).

### Satellite experiments

In order to gain further insight into the As metabolism accompanying biogas production, laboratory scale satellite experiments (1 L scale, batch mode) were carried out. One biogas plant (#1) and one sewage sludge digester (#14) were chosen as basis for these experiments. Slurry was taken from the technical plant and used to fill the satellites (800 mL in case of plant #14, 600 mL + 200 mL oxygen-free water in case of plant #1). In total, six satellite reactors were run per technical plants, in parallels of two. Two of the satellite reactors (satellites 0a and b) were filled with slurry from the technical plants as such and served as controls. In the others the slurries were spiked 24 h after starting the experiment with an additional 2.25 mg L^−1^ (30 µM, satellites 1a and b) or 4.5 mg L^−1^ (60 µM, satellites 2a and b) of arsenite (NaAsO_2_). Nothing else was added in order to avoid influencing the consortia and/or the biogas production by changes in the feed.

**Fig. 4:**
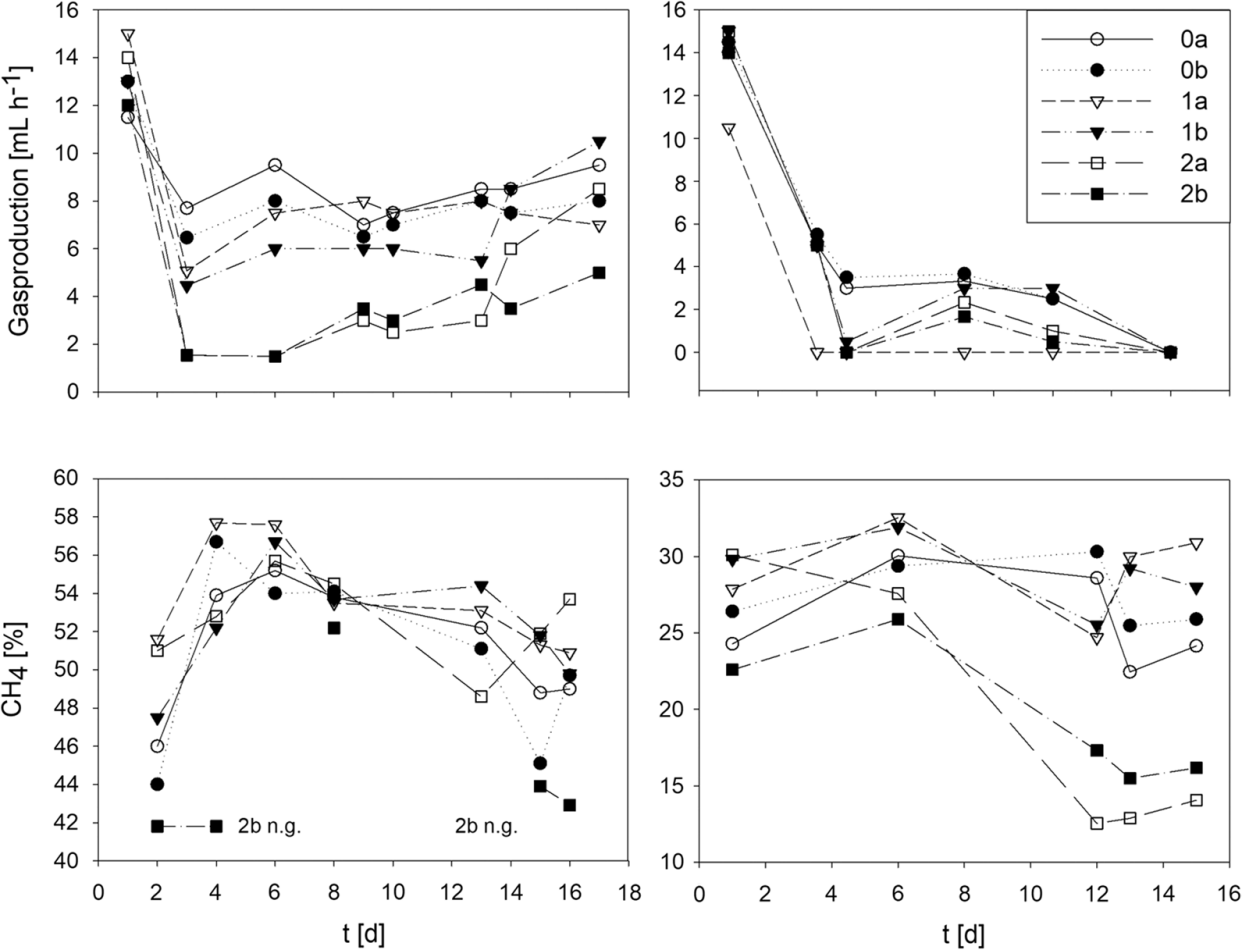
Gas production and CH_4_-content of the gas produced by the satellite reactors. Left: satellites to biogas reactor (technical plant #1), right: satellites to sewage sludge digester technical (plant #14). Satellites 0a and b (controls) contained no additional As, satellites 1a and b contained 30 µM (2.25 mg L^−1^) of additional As, and satellites 2a and b 60 mM (4.5 mg L^−1^) additional As. As was added as NaAsO_2_

The effect of the As addition on the total gas production and relative methane content of the produced gas is shown in Fig. 4. Similar trends are observed for the biogas plant and sewage sludge digester satellites. Within 24 h after starting the satellites, the total produced gas volume halved, while the relative methane content of the gas increased. Afterwards the reactors stabilized and produced a constant amount of biogas for as long as nutrients were available. In case of the sewage sludge digester satellites, the decrease in biogas production was more pronounced and the reactors stabilized at a lower level. Since this is the case also for the controls (no As spike) the most likely explanation is the generally lower nutrient content of the sewage sludge used to start the sewage sludge digester satellites compared to the “richer” sludge from the biogas plant.

The As spike after 24 h had an immediate and dose-dependent effect on the biogas production. Whereas the decrease in total gas production was more pronounced for the spiked reactors than for the controls, the relative methane content of the produced gas tended to be higher, in particular for the intermediate As concentrations (30 µM, satellites 1a and b). During the remainder of the cultivation, most of the contaminated satellites recovered and towards the end of the experiments reached similar biogas production rates as the controls. In general, satellites 1a and b started to recover earlier and more rapidly than satellites 2a and b. In case of the sewage sludge digester satellites, the recovery was less pronounced and most likely overshadowed by the fact that even in the control satellites, the total biogas production ceased almost completely towards the end of the experiments, most likely due to a general lack of nutrients, as already mentioned above.

To investigate whether changes in the microbial community structure accompany the changes in biogas production, the microbial communities from all satellites were subjected to DNA sequencing; the full dataset is available in Additional file 3: Fig. S2. In each case the initial community structure (corresponding to that of the technical plant) used to set up in the satellites was compared to the one established at the end of the process (day 15). It was found that all end-of-culture communities, controls and spiked satellite alike, closely resembled each other, while they differed significantly from the structure initially used to establish the satellites. The reasons for this behavior can at present only be speculated upon. Given the similarities in the development, starvation is most likely not the cause, neither is the transport of the sludge from the technical plant to the laboratory, as initially the communities resembled that of the parent plant. Most likely the fact that the sludges were transferred to a different reactor set up is responsible. A similar effect upon inoculation was also observed in our recent investigation of the startup phase of a technical biogas plant (Weithmann et al. 2016). Thus, the transfer of the slurry to a satellite had a pronounced and reproducible effect on the community structure, whereas the addition—or not—of arsenite had not.

In order to evaluate a possible effect of the arsenite addition on methanogenesis, the acetic acid (HAc) content of the satellites was determined, Table 3. Under anaerobic conditions, HAc constitutes a key metabolite in acetoclastic methane production. The two technical plants used to set up the satellites, namely biogas plant #1 and sewage sludge digester #14, were dominated by archaea from the—often acetoclastic—order of *Methanosarcinales*, the HAc concentration was therefore considered an important indicator for a functional methanogenesis via this pathway in the satellites.

**Table 3:** Acetic acid concentrations in the liquid phase of the satellite reactors [mM]

Satellites to plant	Day	0a	0b	1a	1b	2a	2b
#1 (biogas)	0	5.66	5.66	5.66	5.66	5.66	5.66
	6	0.31	0.25	1.74	2.18	3.55	3.09
	11	< 0.02	0.02	0.43	0.49	4.04	4.35
	16	0.42	0.52	0.07	0.87	0.40	0.95
#14 (sewage sludge)	0	< 0.02	< 0.02	< 0.02	< 0.02	< 0.02	< 0.02
	8	< 0.02	< 0.02	3.25	3.52	4.68	4.86
	16	< 0.02	< 0.02	2.63	< 0.02	9.54	8.87

Satellites 0a/b: controls no additional As spike, satellites 1a/b: 30 mM NaAsO_2_, satellites 2a/b: 60 mM NaAsO_2_

*n.g.* not enough gas produced for analysis

In the control biogas satellites (0a and b) HAc was steadily consumed and increased only towards the end of the 16 d cultivation, which could be indicative of some limitation in acetoclastic methanogenesis towards the end of the experiment. In the corresponding spiked satellites 1a and b (30 µM As), HAc was also consumed, albeit initially at a much lower rate. When the cultures started to recover in terms of biogas production, HAc consumption started to pick up as well. In biogas satellites 2 a and b (60 µM As) HAc was only consumed at the very end of the culture, while intermediately there was even a transitory built up of this important metabolite. The behavior of the sewage sludge satellites was similar. However, in this case the HAc concentration in the control satellites was below 0.02 µM throughout, arguing for efficient methanogenesis via the acetoclastic pathway. For the As spiked sewage sludge satellites, intermediate built up (satellites 1a and b) or steady accumulation (satellites 2a and b) of HAc was observed. The effect was more pronounced in satellite 2a, i.e. the satellite, which recovered least of all in terms of biogas production. Arsenic thus seems to transiently inhibit at least acetoclastic methanogenesis in a dose-dependent manner. This could be due to direct inhibition of the responsible microorganisms, but also due to a competition between As methylation and methanogenesis. Since the microbial community patterns did not change dramatically after As addition, a direct toxic effect is unlikely.

**Fig. 5:**
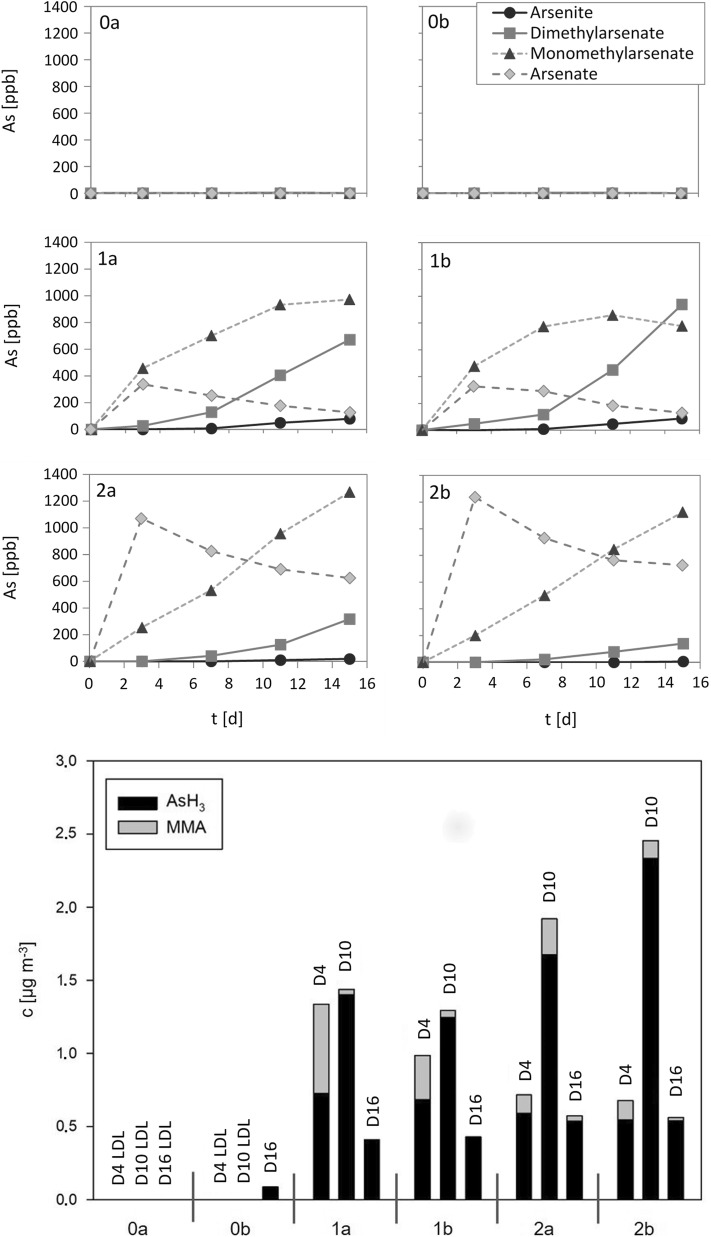
As species in the liquid (top) and gas phases (bottom) of the satellites to the biogas plant (plant #1). Satellites 0a and b (controls) contained no additional As, satellites 1a and b contained 30 µM (2.25 mg L^−1^) of additional As, and satellites 2a and b 60 mM (4.5 mg L^−1^) additional As. As was added as NaAsO_2_. Concentrations of As species in the liquid phase are given as ppb

Figure 5 summarizes the development of the As species in the biogas satellites (liquid phase, produced biogas). As to be expected only very low amounts of arsenite and hardly any other As species are found in the liquid phase of the control satellites (0a and b) to biogas plant #1. In the spiked satellites, a dose-dependent arsenate peak was observed on day 3 accompanied by a steady increase of the concentrations of mono- and later dimethylarsenate in the liquid phase over the duration of the experiment. The concentrations of the volatile As compounds (mainly AsH_3_ and MMA) in the produced gas tended to peak around day 10 of the experiment. Towards the end, AsH_3_ became the dominant As compound in the gas.

**Fig. 6:**
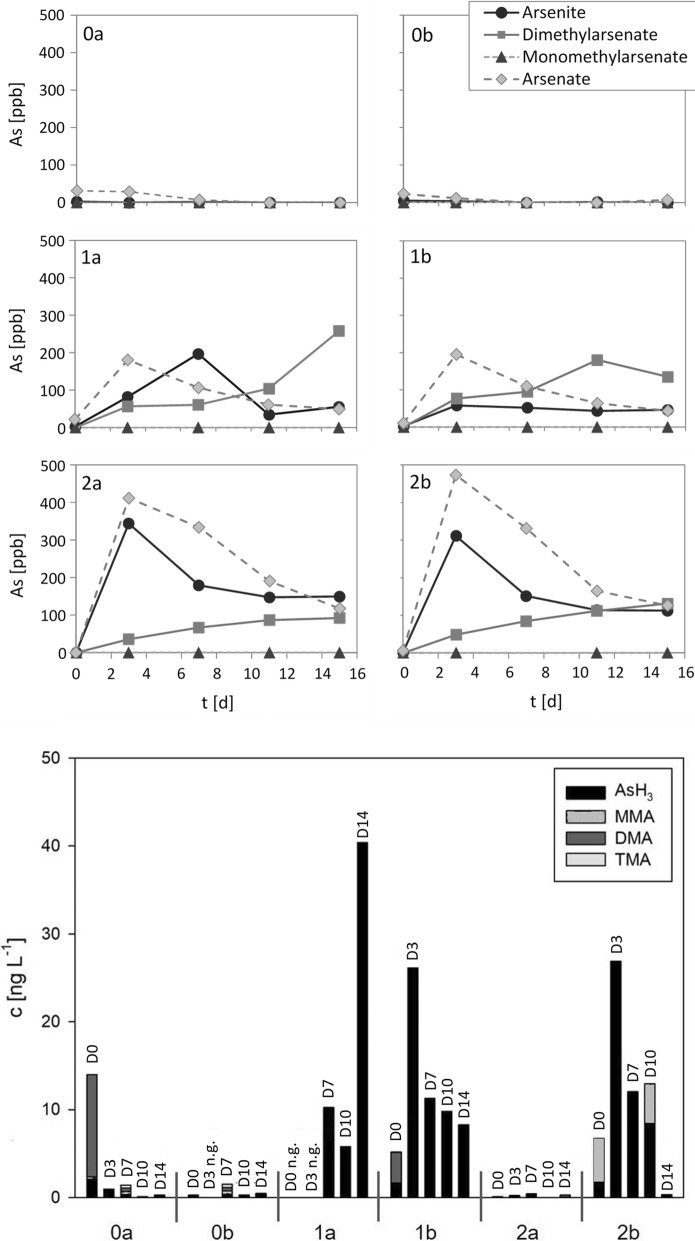
As species in the liquid (top) and gas phases (bottom) of the satellites to the sewage sludge digester (plant #14). Satellites 0a and b (controls) contained no additional As, satellites 1a and b contained 30 µM (2.25 mg L^−1^) of additional As, and satellites 2a and b 60 mM (4.5 mg L^−1^) of additional As. As was added as NaAsO_2_. Concentrations of As species in the liquid phase are given as ppb

The corresponding results for the sewage sludge satellites are summarized in Fig. 6 and show some differences from those of the biogas satellites. As to be expected, there was initially some arsenate present in the liquid phase of the control satellites (0 a and b), which was rapidly degraded, coinciding with a burst of DMA in the corresponding gas samples. Afterwards neither the liquid nor the gas phase of the control satellites contained significant amounts of As. In the spiked satellites, an increase in arsenate was observed in particular during the first 48 h. Concomitantly (satellites 1b, 2a, and 2b) or with some delay (satellite 1a) the concentration of arsenite increased together with the immediate formation of DMA. If anything, only traces of MMA were formed in the sewage sludge satellites. Instead, just as in the corresponding technical plant, AsH_3_ was the dominating As species in most of the gas samples. Satellite 2a did not produce any volatile As species, i.e. As metabolism in that satellite was apparently blocked on the liquid level. Satellite 2b was producing a gas containing mainly AsH_3_ together with some MMA.

## Discussion

This study showed that metabolism and conversion of dissolved into volatile As compounds is ubiquitous in technical biogas plants and sewage sludge digesters. Arsenite in particular was found in the liquid phase of all investigated reactors. This together with the presence of monomethylarsenate as well as some dimethylarsenate in at least two of the reactors (#7, #14), would argue for methylation reactions following the Challenger pathway (Challenger 1945), which comprises a series of oxidation/reduction and concomitant methylation steps. However, alternatives could not be excluded, in particular in case of one of the investigated sewage sludge digester (plant #14), where high amounts of unmethylated arsenate were found in the liquid phase together with mainly AsH_3_ in the gas. It is possible that arsenate was accumulating in this reactor, because the subsequent reactions typical for the Challenger pathway (reduction/methylation) were inhibited or at least rate-limited. Experiments by Cheng and Focht (1979) with different As substrates, comprising arsenite and arsenate species, indicated that direct AsH_3_ formation is possible from various As sources. The authors suggest an alternative to the Challenger pathway for the production of AsH_3_, where methylated arsenates are decarboxylated and directly reduced to AsH_3_. In addition, the formation of AsH_3_ was already shown for pure cultures of methanogenic archaea like *Methanothermobacter thermautotrophicus* and *Methanobacterium barkeri*, found in sewage digesters (Michalke et al. 2000). In our case, *Methanothermobacter thermautotrophicus* was found in plant #10, whereas representatives from the genus methanobacteria, i.e. a genus which includes the species *Methanobacterium barkeri*, were found in plants #12 and #13.

In spite of the presumed As metabolism according to the Challenger pathway, differences can be observed in case of the biogas reactors, both in terms of the efficiency of the conversion and in regard to the specific metabolites produced. For plant #5 in particular, the low As concentration in the liquid phase in view of the composition of the gas phase, argues for an unusually efficient and rapid conversion of dissolved into the volatile A species. Whether these differences can be directly related to differences in the microbial community and in particular the methanogenic archaea or to an inhibitory effect of the higher As burden in the liquid phase of other investigated biogas reactors, is at present not known. The analysis of the archaeal communities in the four plants had shown that plants #1 and #7, but also that of the sewage sludge digester (#14) were dominated by *Methanosarcinales*, i.e. organisms from an order known for acetoclastic methane production. Plant #5 contained mainly archaea from the predominantly hydrogenotrophic order of *Methanomicrobiales*. For details on the community structures of the investigated plants, see Additional file 2: Fig. S1.

It is possible that these differences contribute to the observed variances in the volatile As species produced in these plants. However, in addition to the archaea, bacterial species may also play a role in As methylation and volatilization. Yuan et al. (2008) showed that bacteria containing the *ArsM* or *ArsMC2* genes were able to produce in parallel the volatile species AsH_3_, MMA, DMA and TMA. Huang et al. (2016) described an organism (*Arsenicibacter rosnii*) from the family of *Cytophagaceae*, which was isolated from As contaminated rice paddy soils and converted various arsenite compounds into dimethylarsenate, trimethylarsenate and volatile TMA with high efficiency, without producing AsH_3_ or MMA. However, any extrapolation of these single species results to the behavior of archaeal consortia should be done with caution, since the metabolic networks in the consortia are more divers and redundant and thus more resilient. For instance, while methylation efficiencies of up to 63% of the As present have been shown for entire consortia (Reid et al. 2017), for single strains of *Methanosacine acetivorans* and *Clostridium sp. BXM* efficiencies of only 10 to 13% were found.

Whether elevated and/or changing As concentrations in the range observed in this study have direct consequences for the biogas production in the technical plants is at present unclear, since according to the respective operators all investigated technical plants performed well. The satellite experiments in the presence of As (30 and 60 µM of arsenite) showed negative short-term effects in particular of 60 mM arsenite on the biogas production. However, most satellites recovered quickly in terms of methane and biogas production, as the arsenite was metabolized. The recovery of the biogas production argues for an active decontamination of the environment, most likely through the production of less toxic/inhibitory and/or volatile compounds (Planer-Friedrich et al. 2006). Methylation renders in particular arsenate less toxic (Pakulska and Czerczak 2006), while the volatile As compounds have a low solubility in water compared to arsenites and arsenates and leave the system with the produced gas. Moreover, recovery was quite rapid in all satellites, arguing for a highly active and efficient decontamination metabolism.

Guan et al. (2017) showed that membrane proteins such as ArsB and ACR3 can be used to remove toxic arsenite species from the cells. Alaniz-Andrade et al. (2017) described organisms from the orders of *Bacillus*, *Micrococcus*, and *Acinetobacter*, which could withstand As concentrations as high as 25 mM of arsenite. However, these tolerances were ascribed to adaptation. In our experiments even the satellites to biogas plant #1, which had shown the lowest As content of all investigated plants, showed efficient and rapid metabolization of the added arsenite to less toxic or volatile compounds followed by recovery/stabilization of the biogas production. It is unlikely that this community had previously adapted to elevated As concentrations. Instead, this argues for an active ability for detoxification rather than an adaptation. This is corroborated by the fact that the analysis of the microbial communities showed that the the most pronounced changes in the community structure occurred when the sludge was transferred from the technical plant to the satellite reactors; the As spiking had little, if any, additional effect. This supports the idea of an innate ability to deal with As concentrations in the investigated range, while based on these data no toxic effect can be substantiated.

This is further corroborated by the HAc data and is best illustrated by the reaction of the satellites to biogas plant #1 (low inherent As burden) to the As spikes. Similar to the technical biogas plant, very low amounts of arsenite and hardly any other As species are found in the liquid phase of the unspiked control satellites. In the spiked satellites, on the other hand, hardly any of the added arsenite was actually detected. Instead, a dose-dependent increase of arsenate was observed on day 3 accompanied by a steady increase of the concentrations of mono- and later dimethylarsenate in the liquid phase. The initial built up of the arsenate coincided with the decline in total biogas production and the accumulation of HAc. At the same pace as the concentration of the arsenate decreased, while that of mono- and dimethylarsenate increased, did the biogas production and the HAc consumption rates recover. These findings can be explained by a detoxification mechanism, as identified by Diaz-Bone et al. (2011). Since all other As species increased during the time of recovery, arsenate seems to be responsible for the initial inhibition of the biogas production. Arsenate is a key intermediate in the Challenger pathway, but has also been shown to inhibit at least hydrogenotrophic methane production under laboratory conditions (McBride and Wolfe 1971). Here a similar effect on acetoclastic methane production can be deduced.

In consequence, As concentrations as measured in this study—or even slightly more elevated ones - do not necessarily pose a problem for biogas production. Whether the ensuing contamination of the produced biogas with volatile As species has consequences for the subsequent technical use of the gas as substitute for natural gas, is another question. Even in the most contaminated plants included in this study, the total As content in the gas phase was in the pg NL^−1^ range and thus well below the critical limits for an addition to the gas grid, e.g. in the UK (< 1 ppm, respectively 0.1 µg NL^−1^) (Bright et al. 2011), but also below the safety standard of the arsenic concentration in natural gas (62.5 ng NL^−1)^ given by Xu et al. (2015). The values measured do, however, approach the value, which according to these authors may already cause problems with machinery such as blocked valves, namely 0.01–63.0 pg NL^−1^. Catalyst poisoning is another possibility. As forms stable compounds with Ni, which is part of many industrial catalysts, including those used for the Sabatier process (Sabatier 1910, methane production from CO_2_ using H_2_). Stable NiAs compounds may form in particular in a reaction with AsH_3_. Such reaction will reduce the activity and the lifetime of the catalyst. Similar reactions may occur on the Pd-based catalysts used in cars and buses.

## Supplementary information

**Additional file 1.** Details of the Qiime2 scripts.

**Additional file 2.** Additional electronic material, which provides full dataset of Figure 1 (Qiime2 files).

**Additional file 3.** Additional electronic material (Qiime2 files).

## Data Availability

The data included in the manuscript and additional material, which is saved in the data repository of the University of Bayreuth (https://doubt.uni-bayreuth.de/).
