# Supplementary material for: Arsenic metabolism in technical biogas plants: possible consequences for resident microbiota and downstream units
Source: AMB Express. 2019 Nov 28;9:190. doi: 10.1186/s13568-019-0902-6 (PMC6882981; doi:10.1186/s13568-019-0902-6)
Supplement: Supplementary file 1 — Additional file 1. Details of the Qiime2 scripts. [file 13568_2019_902_MOESM1_ESM.pdf]

Additional file 1

Journal name: AMB Express

Manuscript Title: Arsenic metabolism in technical biogas plants: possible consequences for resident microbiota and downstream units

Authors: Nicolas Weithmann[1], Stanislava Mlinar[1], Frank Hilbrig[1], Samer Bachmaf[2], Julia Arndt[2], Britta Planer-Friedrich[2], Alfons R. Weig[3], Ruth Freitag[1,*]

[1]Process Biotechnology and Centre for Energy Technology (ZET), University of Bayreuth, Universitätsstraße 30, 95447 Bayreuth, Germany

[2] Department of Environmental Geochemistry, Bayreuth Centre for Ecology and Environmental Research (BayCEER), University of Bayreuth, Universitätsstraße 30, 95447 Bayreuth, Germany

[3] Genomics and Bioinformatics, University of Bayreuth, Universitätsstraße 30, 95447 Bayreuth, Germany

* Corresponding author: E-Mail address: ruth.freitag@uni-bayreuth.de; Phone number: +49(0)921557371, Fax: +49 (0)921557375

The following supplementary material can be obtained from data repositories at the University of Bayreuth, Germany:

Additional file 2: https://doi.org/10.15495/do_ubt_953

Additional file 3: https://doi.org/10.15495/do_ubt_954

The supplementary materials represent taxonomic classifications of the data analyzed by the Qiime2 next-generation microbiome bioinformatics platform (https://qiime2.org/) as described in the main text.

After download of the supplementary material, the classification files (.qzv) can be viewed by the Qiime2view interface https://view.qiime2.org/;

Instructions and examples how to use the Qiime2 interface are available at https://view.qiime2.org/.
